# *Figla*-*Cre* Transgenic Mice Expressing Myristoylated EGFP in Germ Cells Provide a Model for Investigating Perinatal Oocyte Dynamics

**DOI:** 10.1371/journal.pone.0084477

**Published:** 2014-01-06

**Authors:** Ruei-Shiuan Lin, Maria Jimenez-Movilla, Jurrien Dean

**Affiliations:** Laboratory of Cellular and Developmental Biology, National Institute of Diabetes and Digestive and Kidney Diseases, National Institutes of Health, Bethesda, Maryland, United States of America; McGill University, Canada

## Abstract

FIGLA (Factor in the germline, alpha) is a bHLH transcription factor expressed abundantly in female and less so in male germ cells. Mice lacking FIGLA do not form primordial follicles in the ovary and females are sterile, but there is no obvious phenotype in males. Using the *Figla* promoter to express Cre recombinase, we have established *mEGFP/mTomato* reporter mice with green germ cells and red somatic tissue. These mice were crossed into the *Figla* null background to accelerate perinatal oocyte loss. Live imaging of cultured newborn ovaries provides evidence that few oocytes egress and the vast majority disappear within the confines of the ovary. Although a cohort of mobile, phagocytic cells was observed, macrophage depletion in *Csf1^op/op^* mice did not affect oocyte loss. Investigations with TUNEL assays and caspase inhibitors suggest that apoptosis plays a role in the perinatal loss of oocyte in female mice. These results establish the utility of *Figla-EGFP/Cre; mTomato/mEGFP* in investigating germ cell dynamics in prepubertal mice.

## Introduction

Folliculogenesis begins just prior to birth when ovarian somatic cells invade oocyte cysts to form primordial follicles in which individual germ cells are encased by a single layer of granulosa cells [1]. During the two days following parturition, there is a substantial reduction in the number of primordial follicles [2] and only those that survive are available during the female's reproductive life. The advantage(s) of this early loss in germ cells has perplexed investigators and explanations range from limits in nutrient supplies to quality control for meiosis and proper follicle formation [3]–[5]. Further uncertainty arises from an inadequate understanding of the physiological basis of this programmed cell death which has been attributed variously to apoptosis, autophagy and oocyte egression or shedding from the ovary.

Mice deficient in *Casp2* (encodes caspase 2) form more and *Bcl2* or *Bcl-x* (anti-apoptosis factors) null ovaries form fewer primordial follicles suggesting a role for apoptosis in oocyte loss during embryonic development [6]–[8]. *Bax* (a pro-apoptotic factor) null mice also exhibit increased number of primordial follicles in newborn ovaries, but this reflects a larger reservoir of oogonia that accumulate during gonadogenesis [9]. Based on increased abundance of lysosomes early in folliculogenesis, autophagy was initially suggested as a potential cause of oocyte-loss [10]. However, newborn ovaries from *Atg7* (an E1-like ligase required for lipid conjugation of LC3) null and *Becn1* (required for vesicle formation of autophagosomes) heterozygote newborn ovaries have increased germ cells suggesting that autophagy promotes germ cell survival rather than programed cell death [11], [12]. Although egression during folliculogenesis has been observed by several groups [13]–[15], the extent to which it accounts for perinatal oocyte loss is unknown.

Factor in the germ line, alpha, FIGLA, is a basic helix-loop-helix transcription factor that up-regulates female-specific and down-regulates male-specific genes during oogenesis [16]–[18]. Although male mice are not overtly affected, its ablation in female mice precludes formation of the primordial follicle and results in massive, perinatal oocyte loss. Within two days after birth virtually all female germ cells have been lost and adult female mice are sterile [19]. We have sought to exploit this accelerated time schedule for the loss of germ cells to establish models for investigating the molecular mechanisms underlying the normal physiological process of perinatal depletion of oocytes.

Using transgenesis, we have established a mouse line, *Figla-EGFP/Cre*, that expressed EGFP and Cre recombinase beginning at E14.5 in female germ cells. By crossing this line into m*Tomato*/m*EGFP* reporter mice, we obtain germ cells with membrane-bound EGFP in gonads expressing m*Tomato* in their somatic compartment. Using these mice either before or after crossing them into the *Figla* null line, we have established an organ culture system to investigate early folliculogenesis. As a proof of principal of this investigative tool, we observe the disappearance of oocytes in the perinatal ovary over several days of culture. Results indicate that oocytes do not escape, but are programed to die within the ovaries. Macrophages are not required and oocyte death results from caspase-dependent apoptosis.

## Results and Discussion

### Characterization of *Figla-EGFP*/*Cre* Mice

**Figure 1:**
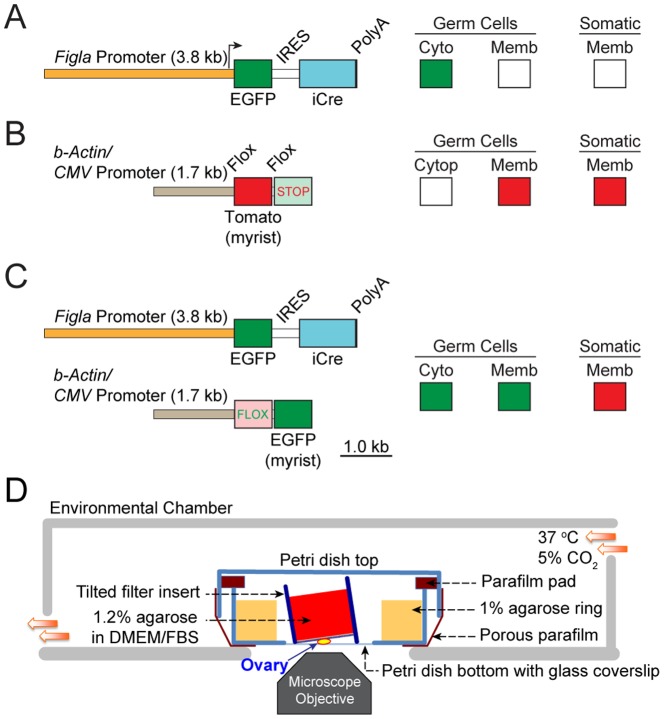
Gonad-specific expression of Cre in the *Figla-EGFP/Cre*; *mTomato/mEGFP* mice. (A) Transgene designed for bicistronic expression of both EGFP and Cre under the control of *Figla* promoter (3.8 kb) was used to generate the *Figla-EGFP/Cre* transgenic mice. EGFP is present in the cytoplasm (Cyto) of germ cells. (B) Reporter mice with floxed *mTomato* (myristoylated Tomato) and *mEGFP* driven by the universal β-actin/CMV promoter (1.7 kb). *mTomato* is expressed in somatic and germ cells where it is anchored to the membrane (Memb). (C) Mice with both (A) and (B) transgenes express mTomato in all somatic cells including those in the gonads. Both cytoplasmic (from A) and membrane bound (from B) EGFP are present in germ cells in double transgenic mice (dTg). (D) Isolated mouse ovaries were imaged by confocal microscopy using a 20× objective and a petri dish with a glass coverslip. The ovary was placed under a tilted Millicell Cell Culture Insert with a filter bottom that was filled with 1.2% agarose (DMEM/FBS) and surrounded a ring of agarose gel to maintain moisture. The cover and petri dish were tightly opposed with a tight-fitting parafilm pad and wrapped in parafilm with pores allowing gas exchange with an outside environmental chamber (37 °C, 5% CO_2_).

**Figure 2:**
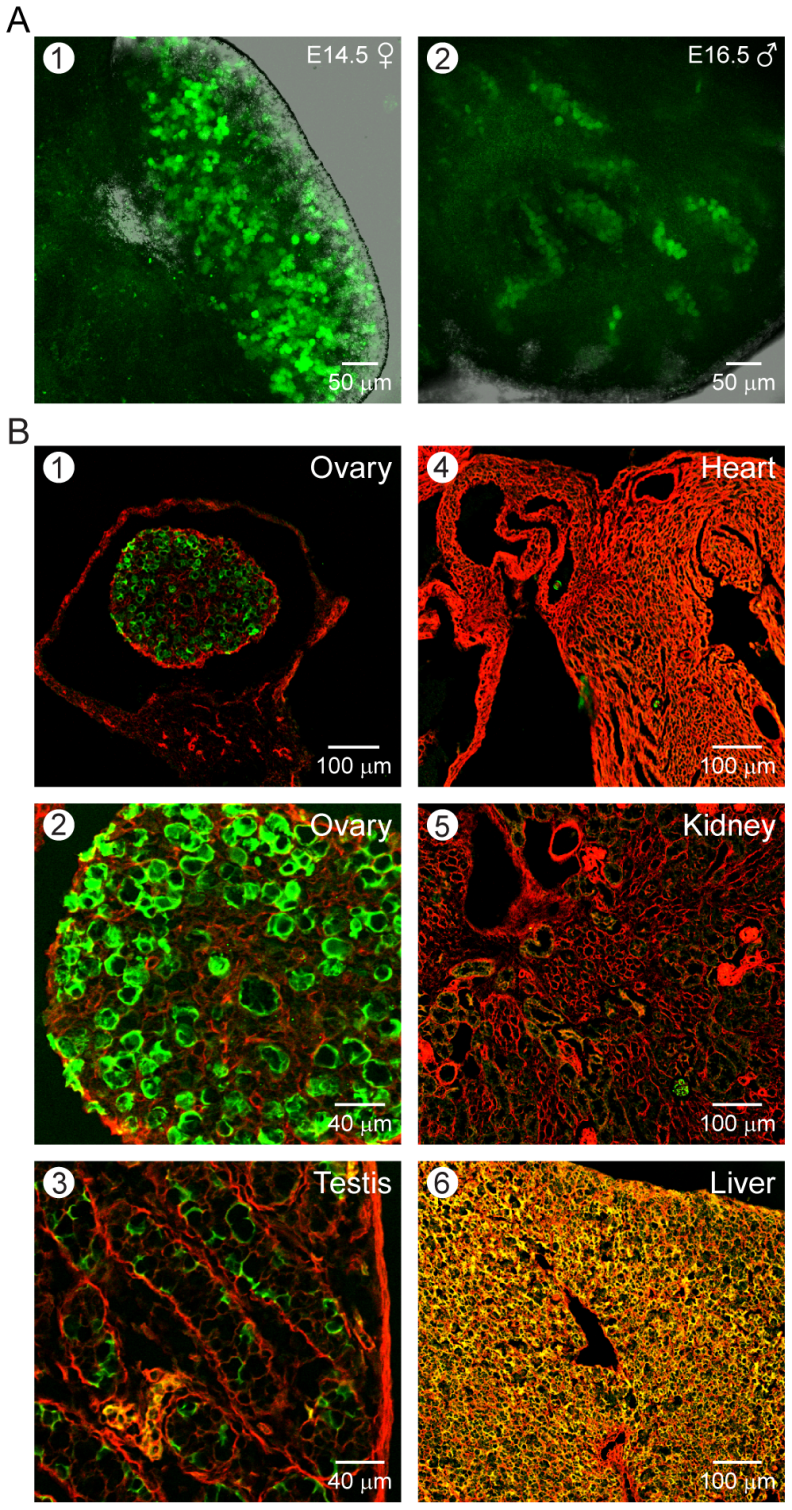
EGFP expression in *Figla-EGFP/Cre* transgenic mice. (A) Female (1) and male (2) embryonic gonads at E14.5 and E16.5, respectively, were dissected from hemizygous *Figla-EGFP/Cre* transgenic mice and whole-mount images were obtained by laser scanning confocal microscopy. (B) After crossing into *mTomato/mEGFP* reporter mice, EGFP was detected in either ovary (1,2) or testis (3) of newborn mice where it was more intense at the membrane than in the cytoplasm. It was not detected in heart (4), kidney (5) or liver (6). Strong auto-fluorescence was present in hepatic tissues.

We established transgenic mice with EGFP and Cre recombinase under control of a 3.8 kb *Figla* promoter to ensure germline-specific gene expression. Both male and female hemizygous animals from two founder lines appeared healthy and fertile. The DNA encoding EGFP and Cre were separated by an internal ribosomal entry site (IRES) to ensure independent translation of the two proteins (Fig. 1A). Transcription factor FIGLA is expressed in female gonads as early as E13.5, just before the onset of meiosis, and the abundance of its transcripts is maximal around birth [16]. Compared to female germ cells in which strong EGFP signals could be detected as early as E14.5, male germ cells showed weak green fluorescence at E16.5 (Fig. 2A). Thus, EGFP and Cre recombinase driven by *Figla* promoter can be used for conditional ablation of genes near the onset of meiosis and complement the recently published *Spo11*-*Cre* transgenic mouse [20] as efficient tools to study female germ cells during embryogenesis.

To investigate the additional potential for tracking germ cells during gonadogenesis, the *Figla-EGFP/Cre* mice were crossed into an *mTomato/mEGFP* reporter mouse line to follow oocyte development and confirm correct expression of Cre recombinase. *mTomato/mEGFP* mice carry ubiquitously expressed myristoylated Tomato fluorescent protein (Fig. 1B). In the presence of Cre recombinase, the gene encoding mTomato is excised and instead, the downstream gene encoding myristoylated EGFP (*mG*) is expressed [21]. Thus, in double transgenic mice (*Figla-EGFP/Cre*; *mTomato/mEGFP*) which were designated dTg, germ cells expressing Cre recombinase have EGFP fluorescence both in the cytosol and on the plasma membrane (Fig. 1C). Somatic cells remain red because of the continued expression of *mTomato*. We observed that in dTg mice, the myristoylated EGFP fluorescence was brighter than cytosolic localized EGFP (Fig. 2B, panel 2). As expected, the expression of Cre recombinase, as assessed by plasma membrane localized EGFP fluorescence, was observed in newborn ovaries and was absent in brain, heart, liver, or kidney (Fig. 2B). Unexpectedly, faint EGFP signals were also observed in testes, seemingly in male germ cells.

**Figure 3:**
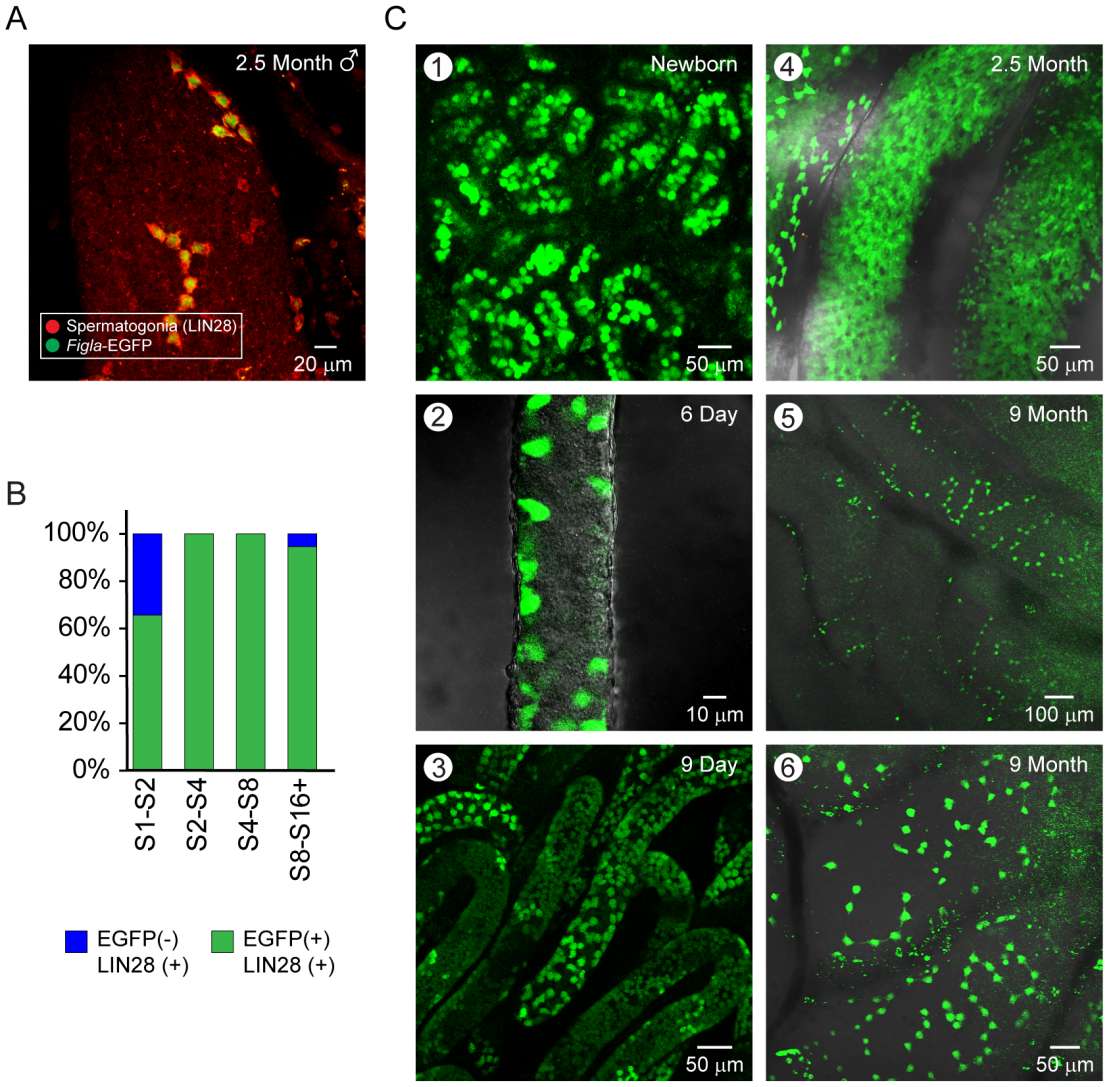
EGFP expression in *Figla-EGFP/Cre* transgenic testes. (A) EGFP is expressed early in undifferentiated spermatogonia identified by reactivity with antibodies to LIN28 in testes from 2.5 month old *Figla-EGFP/Cre* male mice. Z projections were acquired by scanning fixed whole mount preparations and collapsed onto a single plane to determine the length of chained spermatogonia. Only chained clusters with well-defined ends were used for quantification. (B) Bar graph of data in (A) divided among chains of A-aligned spermatogonia. Length of spermatogonia cell chains indicated numerically (e.g., S2-S4). (C) EGFP expression detected in gonocytes of newborn (1) and 6 days old testis (2) as well as undifferentiated spermatogonia of 9 days (3), 2.5 months (4), and 9.5 months old testis (5, 6). In newborns, gonocytes were present in the center of seminiferous tubule (1). By six days after birth, they had migrated to the periphery (2) and had proliferated by day 9 (3). In adult testes (4–6), EGFP was expressed strongly in chained spermatogonia.

Upon further investigation, strong green fluorescence was observed in gonocytes (primordial germ cells) [22] present in the lumen of seminiferous tubules of mouse testes (Fig. 3A). These EGFP expressing cells also expressed Lin28, a marker of spermatogonia [23] and were grouped in strings of intercellularly connected cells as previously described [24]. Although all female germ cells were green in newborn ovaries (co-staining with antibodies to Mvh/DDX4), not all gonocytes in the newborn testes switched to green fluorescence suggesting either heterogeneous expression or a modest expression of Cre recombinase driven by the *Figla* promoter in embryonic male germ cells (Fig. 3B).

To analyze *Figla* promoter activity in greater detail, we isolated gonads from both sexes of *Figla-EGFP/Cre* and dTg mice at E12.5, E14.5, or E16.5. Compared to female germ cells in which strong EGFP signals could be detected as early as E14.5, male germ cells showed weak green fluorescence at E16.5 (Fig. S1). It has been reported that there might be a delay of Cre recombinase catalyzing DNA recombination after Cre expression [25]. However, in *Figla-EGFP/Cre; mTomato/mEGFP* mice, the expression of myristoylated EGFP was at the same stage as *Figla* promoter activation (cytosolic EGFP in *Figla-EGFP/Cre* mice) in E14.5 females and E16.5 males suggesting efficient DNA recombination occurred without delay (Fig. S1, insets).

### *Figla* Is Expressed in Undifferentiated Spermatogonia

Initial reports on FIGLA emphasized its expression in female germ cells and role in regulating oocyte-specific target genes to maintain sexual identity during post-natal gametogenesis [17]–[19]. However, low levels of *Figla* transcripts were detected in testicular tissue by RT-PCR [16] and more recently in spermatogonia enriched cDNA libraries [26]. In retrospect, small amounts of FIGLA protein are present in adult testes and were detected in a functional, gel-mobility shift assay [19], [27].

During spermiogenesis, the male haploid genome is repackaged from somatic histones onto transition proteins and then onto protamines where it remains transcriptionally quiescent prior to fertilization [28]. These small basic proteins form intermolecular disulfide bonds and account for the dense nucleus of the mature sperm. However, depending on the species, 1–4% of the sperm DNA remains packaged on somatic nucleosomes [29]–[31]. Some human genes that are packaged on epigenetically marked (H3K4me3) nucleosomes in sperm play important roles in pre-implantation development [32], [33], but whether this pre-positioning affects their expression has yet to be determined. In addition, there are a number of genes expressed in spermatogonia (e.g., *KIT*, *SALL4*, *NANOS3*, *LIN28A*, *GFRA1*, *ZBTB16*) that escape being packaged by protamines and remain in H3K27me3 marked nucleosomes, a permissive environment for gene expression. The promoter of *Figla* similarly escapes repressive packaging in chromatin which may account for its expression in spermatogonia. However, the function of FIGLA in male gonad remains unclear and as no obvious defects in *Figla* null male fertility or spermatogenesis were observed in the present or in a previous report [19].

**Figure 4:**
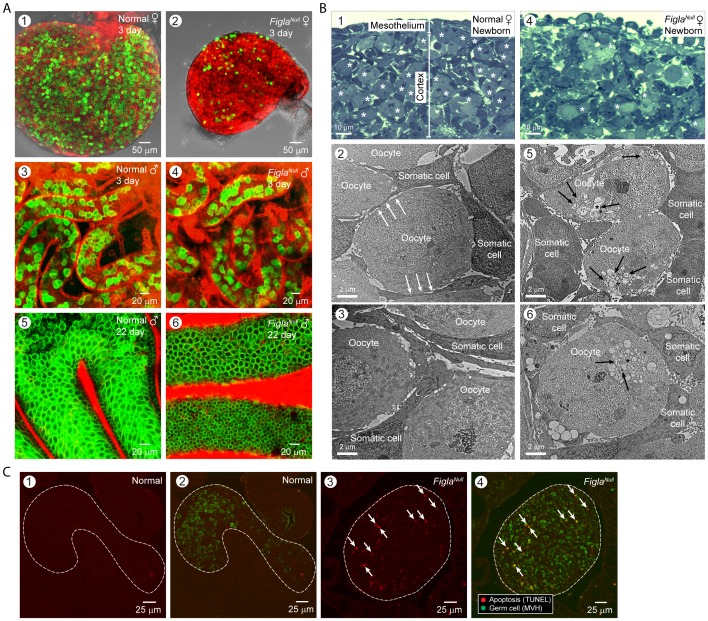
Germ cell development in postnatal gonads of *Figla* null; *Figla-EGFP/Cre* mice; *mTomato/mEGFP*. (A) Ovaries (1,2) or testes (3–6) were collected from 3 day (1–4) or 22 (5,6) d/o normal (1,3,5) and *Figla* null; *Figla-EGFP/Cre* mice; *mTomato/mEGFP* (2,4,6) mice. Unlike the dramatic germ cell reduction observed in ovaries (1,2), spermatogenesis in *Figla* null testes remained normal (3–6). (B) The cortex of newborn ovaries was imaged by light (1,2) and electron microscopy (3–6) of normal (1–3) and *Figla* null (4–6) mice. Many fewer oocytes (white asterisks) were present in *Figla* null (4) compared to normal (1) ovaries. Somatic (pregranulosa) cells extend processes (white arrows) between adjacent oocytes in normal newborns (2) that were short and dissociated from adjacent oocytes or absent in *Figla* null ovaries (5). The cytoplasm of *Figla* null oocytes (6) had numerous dispersed vesicles (black asterisks), many of which contained cytoplasmic structures in different phases of degradation including membrane debris (black arrows). (C) Germ cell apoptosis was analyzed in normal (1,2) and *Figla* null (3,4) ovaries using TUNEL (red) alone (1,3) or combined (2,4) with anti-Mvh (DDX4) antibody (green). Arrows indicate apoptotic oocytes with co-localization of the two signals (yellow). Ovarian tissue is outlined by a dotted line.

To observe reporter gene expression in *Figla-EGFP/Cre* mice, tubules from post-natal day 0.5 (P0), P6, and P9 were collected for whole mount confocal microscopy where EGFP was readily detected in gonocytes, primitive spermatogonia A and mature spermatogonia A/B (Fig. 3C). In 22 d/o testes, cells with the most intensive fluorescence were located at the periphery of the seminiferous tubule identifying those cells as spermatogonia (Fig. 4A, panel 5). To confirm spermatogonia expression of EGFP in adult transgenic mice, we collected testes from 2.5 and 9 month old mice (Fig. 3C, panels 4–6). When whole mount testes were examined, those intensively labeled cells were found adjacent to the vesicular system and existed either as a single cell or as aligned, interconnected cells numbering 2, 4, or 8 (Fig. 3C, panels 5,6) consistent with earlier observations [24].

In the current study, EGFP was also observed in more differentiated male germ cells which could reflect the stability of the EGFP expressed earlier in spermatogenesis. We note that EGFP is observed in the cytoplasm of male germ cells through all stages of spermatogenesis in mice expressing the single *Figla-EGFP/Cre* transgene. From these observations, we conclude that *Figla* is expressed in undifferentiated spermatogonia and that *Figla-EGFP/Cre*; *mTomato/mEGFP* dTg mice provide a useful system for their imaging.

### Live Imaging of Oocyte Loss in the Perinatal Ovary

The process of perinatal oocyte death has been described in several reports. In mice, it has been shown that oocyte numbers decreases around fifty percent between E18.5 and P2 [3]. In some genetic ablation mouse models, mutant oocytes vanish within few days after birth without inducing inflammation [19], [34]. In addition, although apoptosis has been suggested to be the main mechanism for oocyte disappearance, the number of apoptotic oocytes detected by the steady-state apoptotic index could not account for the total loss number. Thus, multiple-mechanisms mediated early oocyte death has been suggested [10].

Taking advantage of strong, membrane limited *mTomato/mEGFP* expression, we used live imaging of the dTg mice to track oocytes during primordial follicle formation. Because early folliculogenesis is hormone independent [35], we designed an *in vitro* organ culture that allowed us to observe cellular events in newborn ovaries for up to 7 days (Fig. 1D). Confocal Z projections obtained at 10 or 15 min internals documented that cultured newborn ovaries from the *Figla-EGFP/Cre; mTomato/mEGFP* mice recapitulated the process of primordial follicle formation. Oocyte cysts break down to liberate individual MI-arrested oocytes some of which form primordial follicles while others seemingly disappear (Video S1 and S2). Few oocytes were observed egressing from the surface of the ovaries and the vast majority of oocytes were lost within the gonad (Video S3). Unexpectedly, we observed a cohort of highly mobile, macrophage-like cells among oocyte clusters (Video S4) that appear to phagocytize individual germ cells.

**Figure 5:**
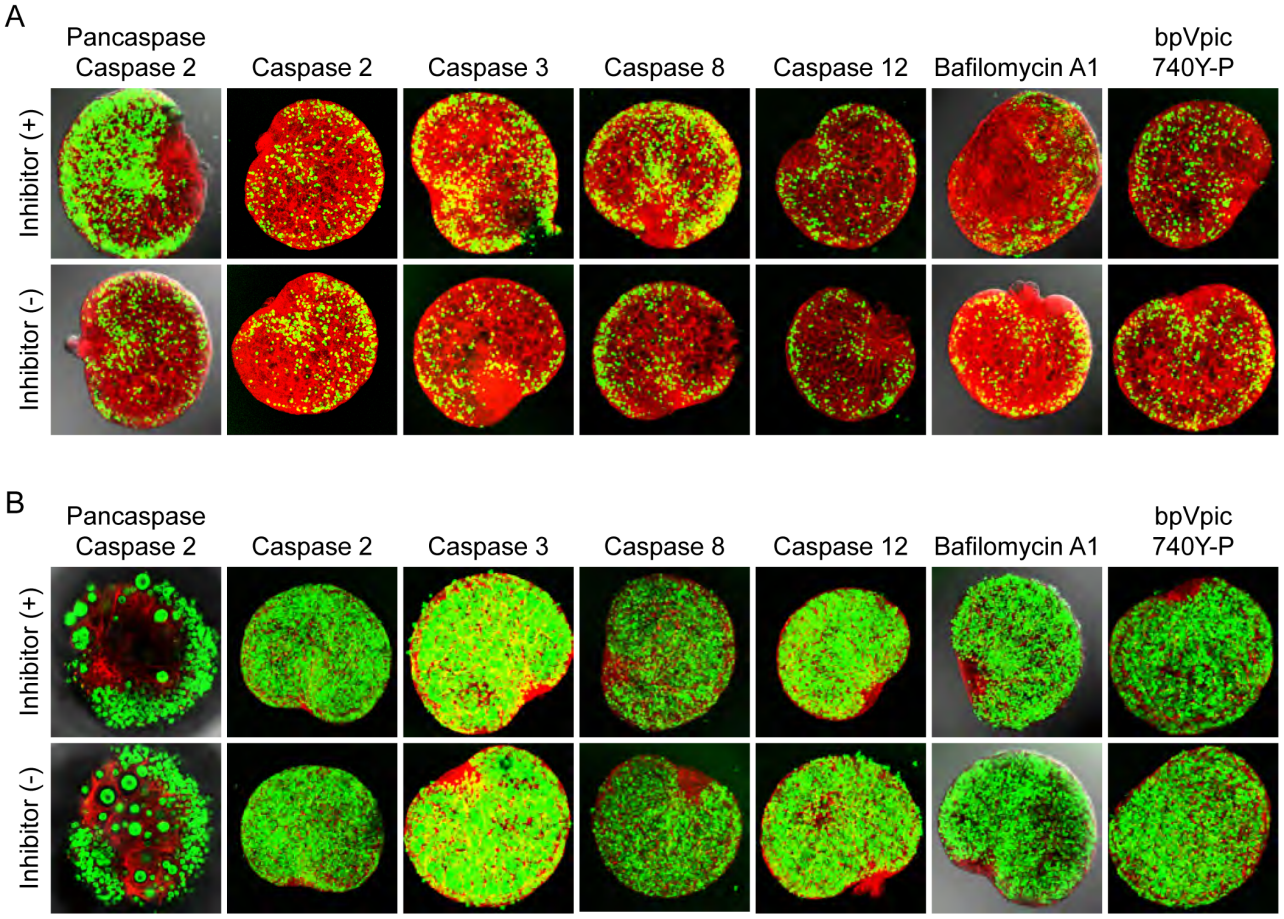
Oocyte apoptosis in *Figla* null ovary is caspase dependent. Newborn ovary pairs from *Figla* null (A) and normal mice (B) were cultured *in vitro* with or without 100 µM caspase inhibitors (pan caspase: Z-VAD-fmk; caspase 2: Z-VDVAD-fmk; caspase 3: Z-DEVD-fmk; caspase 8: Z-IETD-fmk; caspase 12: Z-ATAD-fmk), 2 µM Bafilomycin A1, or 100 µM bpV(pic) plus 500 µg/mL 740Y-P for 2 (except for pancaspase which was for 7) days at 37 °C. (A) Oocyte loss in *Figla* null ovaries was significantly inhibited in the presence of the pancaspase inhibitor and to a lesser extent with inhibitor for caspase 8 and 3. (B) The presence of inhibitors did not morphologically or physiologically affect normal oocytes. In these assays, only ovary pairs with comparable numbers of healthy oocytes were selected for *in vitro* culture. The hilum of each ovary was juxtaposed on the filter (Fig. 1D) and images were obtained by laser scanning confocal microscopy from the opposite side of the ovary. Z projections of EGFP fluorescence intensity were collapsed into a single plane to visualize the number of germ cells before and after treatment with inhibitors. Scale bar, 100 µm.

To better observe oocyte degradation, we sought to accelerate the process by examining ovaries from *Figla* null mice which feature a massive loss of oocytes in the first two days after birth (Fig. 4A, panels 1,2; Fig. 4B, panels 1,4). A similar loss was not observed in male germ cell in testes isolated from normal and *Figla* null male mice at 3 and 22 days after birth (Fig. 4A, panels 3–6). The disappearance of female germ cells is associated with a failure to form primordial follicles and oocytes do not form junctions with somatic cells (Fig. 4B, panels 2,3,5,6). Prior to loss, the oocytes appear morphologically and developmentally normal [19]. Oocyte apoptosis was readily detected in the newborn *Figla* null ovaries by TUNEL assays and was more than 4-fold enhanced in *Figla* null (160±31/A.U., n = 4) compared to normal (38±8/A.U., n = 3) ovaries (Fig. 4C). With a concentration sufficient to inhibit irradiation-induced apoptosis, pan-caspase inhibitors efficiently reduced *Figla* null oocyte apoptosis *in vitro* (Fig. 5A) compared to normal oocytes (Fig. 5B). To a lesser extent, inhibitors for caspase 8 and 3 reproducibly enhanced survival of *Figla* null oocytes while inhibitors for caspase 2 or 12 had little effect. Electron microscopy detected what appeared to be autophagic vesicles in *Figla* null oocytes (Fig. 4B, panels 5,6), but bafilomycin A1, an inhibitor of autophagy [36] had no significant effect, as was the case for bpV(pic)/740Y-P, activators of the PI3K-Akt-FOXO3 pathway [37] (Fig. 5). Although apoptosis appeared a significant determinant of oocyte loss in *Figla* null mice, its affect in normal mice was not documented in these studies.

**Figure 6:**
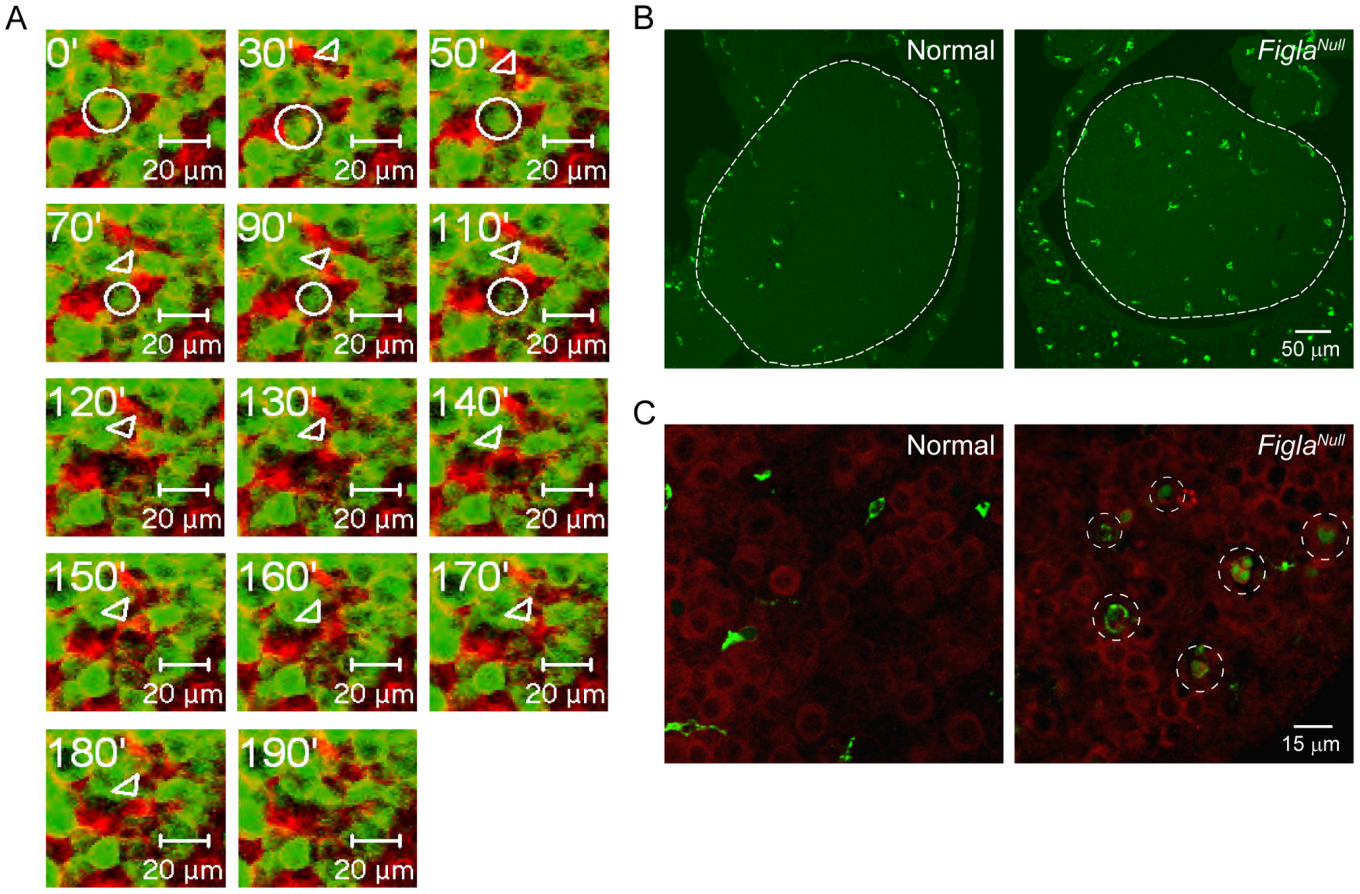
Macrophages involved in clearance of dead oocytes in normal and *Figla* null ovaries. (A) Time-lapse recording of a normal *Figla-EGFP/Cre*; *mTomato/mEGFP* newborn ovary captured a destruction process of an oocyte (circle). A somatic cell (arrowhead) closely associates with the oocyte and appears to phagocytize the remains of the degrading oocyte. (B) Normal or *Figla* null newborn ovary sections were immuno-stained with F4/80, a macrophage specific marker. Ovarian tissue is outlined by dotted line. (C) Oocytes and macrophages were stained by Mvh (red) or F4/80 (green), respectively. Macrophage-engulfed degrading oocytes were observed in *Figla* null (dotted circles), but not normal ovaries.

We also investigated a potential role for motile, macrophage-like cells that were observed in *Figla* null ovaries that experience massive oocyte loss (Video S2). These cells were attracted to degraded oocytes which, on occasion, they phagocytosed in cultured normal ovaries (Fig. 6A). Although unable to confirm their presence by live imaging, they were identified using F4/80, a pan-macrophage marker, in fixed ovaries from normal (139±17/A.U., n = 4) and *Figla* null (199±41/A.U., n = 3) mice (Fig. 6B) and some had engulfed oocytes in *Figla* null ovaries (Fig. 6C). To further resolve this issue, we examined oocyte loss in 2 d/o ovaries from normal and *Csf1^op/op^* mice (Fig. S2, panel A) that are deficient in macrophages [38]. However, no significant difference in the number of degenerating pyknotic oocytes were observed between normal and *Csf1^op/op^* mutant ovaries (Ρ<0.076, n = 3) even after crossing the *Csf1^op/op^* mice into a *Figla* null background to accentuate oocyte loss (Fig. S2, panel B). Taken together these observations suggest that rather than a primary role in in oocyte loss, macrophages may play a secondary role in oocyte homeostasis during perinatal follicle formation.

## Conclusions

To our knowledge, this is the first live imaging record of primordial follicle formation during mammalian ovarian folliculogenesis. In these studies we were able to observe ovarian cyst breakdown, oocyte growth and subsequent germ cell degeneration. Although their identities remain elusive, a cohort of mobile somatic cells actively associates the adherent oocytes within cysts and with degenerating oocytes. We speculate that their involvement in oocyte degeneration during folliculogenesis is primary for the rapid cleansing of the detritus following oocyte loss.

Oocyte egression and autophagy, were not observed in our live imaging and *in vitro* ovary culture experiments and caspase-dependent mechanisms for oocyte loss seem most likely, at least in *Figla* null mice. It has been reported that caspase 2, but not caspase 3, is responsible for oocyte apoptosis in normal mice [7], [39] and that caspase 7 may play a role during embryonic germ cell loss [40]. Our results indicate that the apoptosis observed in *Figla* null ovaries [19] involves caspase 8 and 3 which may act in concert or sequentially [41], but the trigger mechanism(s) remain to be determined. From these observations, it seems likely that different caspases can mediate oocyte loss depending on the period of development and experimental conditions. Thus, our findings are consistent with current understanding that apoptosis mediates the oocyte loss in perinatal ovaries and provides a transgenic system for live imaging of peri- and post-natal dynamics of male and female germ cells in normal and genetically altered mice.

## Materials and Methods

### Ethics Statement

Experiments with normal and transgenic mice were conducted in accordance with the recommendations in the Guide for the Care and Use of Laboratory Animals of the National Institutes of Health. The protocol was approved by the NIDDK Animal Care and Use Committee of the Division of Intramural Research in compliance with the guidelines of the Animal Care and Use Committee of the National Institutes of Health (Animal Welfare Assurance #A4149-01).

### Materials

All chemicals were obtained from Sigma-Aldrich (St. Louis, MO) except for those described otherwise. Vectors containing EGFP and IRES were obtained from Clontech (Mountain View, CA); iCre from Addgene (Cambridge, MA) and the 3.8 kb *Figla* promoter from a BAC clone [19]. Primary antibodies include: rabbit anti-Mvh (DDX4) from Abcam (Cambridge, MA); rat anti- F4/80 from AbD Serotec (Raleigh, NC). Secondary antibodies include: Alexa466 coupled donkey anti-rat from Life Technologies (Grand Island, NY); Dyelight coupled goat anti-rabbit from Roche Applied Science (Indianapolis, IN); goat anti- rabbit and goat anti-rat from Molecular Probe (Grand Island, NY). General or specific caspase inhibitors and bpV(pic) were obtained from EMD Millipore (Billerica, MA). 740Y-P was from Tocris (Bristol, UK).

### Establishment of Transgenic Mice

A 3.8 kb DNA fragment containing the *Figla* promoter and partial sequence of the first exon was subcloned into the pEGFPLuc vector (Clontech) from which the CMV promoter had been removed. After digestion with *Sac1* and *Mfu1* to remove the luciferase coding sequence, the resultant vector was ligated with two PCR-amplified fragments: IRES flanked 5′ by *Sac1* and 3′ by *Sal1* sites; and iCre flanked 5′ by *Sal1* and 3′ by *Mfu1* sites. After digestion of the final construct with *Spe1* and *Mfu1*, the 6.4 kb DNA fragment was gel purified and injected into the pronuclei of 1-cell zygotes to establish transgenic mice designated *Figla-EGFP/Cre*. Two founders that passed the transgene through their germline were maintained as homozygotes with normal fertility and no adverse phenotype. The B6;C3F *a/a-Csfl^op^*/J mice lacking macrophages and the *Gt(ROSA)26Sor^tm4(actb-tdTomato-EGFP)Luo^*/J reporter mice (abbreviated *mTomato/mEGFP ^+/+^*) were obtained from The Jackson Laboratory (Bar Harbor, ME). *Figla^tm1Dean^* mice were obtained from the NIH colony.

*Figla-EGFP/Cre* and *mTomato/mEGFP ^+/+^* mice were crossed to establish the reporter mouse line and the *Figla-EGFP/Cre; mTomato/mEGFP ^+/+^*mice were then crossed into the *Figla* null background [19], Male *Figla-EGFP/Cre^+/+^*; *Figla^−/−^* and female *mTomato/mEGFP ^+/+^*; *Figla^+/−^* mice were mated to obtain *Figla-EGFP/Cre; mTomato/mEGFP; Figla^−/−^* mice. *Csf1^op/op^*; *Figla* ^−/−^ mice were generated from *Csf1^op/+^*; *Figla* ^−/+^ parents. The DNA sequences of primers used for genotyping of isolated tail DNA are presented in Table S1.

### Tissue Expressions of the Transgenes

Organs from newborn *Figla-EGFP/Cre; mTomato/mEGFP* mice were fixed in 4% paraformaldehyde in PBS, pH 7.4 at room temperature for 1 hr and stored at 4 °C overnight. Fixed samples were cryosectioned (5 µm) and stored at −20 °C.

### In Vitro Ovary Culture and Time-lapse Confocal Microscopy

The whole-mount immunofluorescence was performed as described [23]. Ovaries were carefully removed at postnatal day 0.5 (P0.5), immersed in limited amounts of Weymouth medium/10% FBS (Life Technologies), and cultured in a Millicell Cell Culture Insert with the hilum against the membrane filter (PICMORG50, EMD, Millipore). The whole culture was then placed in a moisture chamber in the presence of 5% CO_2_ at 37 °C [35]. For experiments with inhibitors, pairs of ovaries with comparable numbers of oocytes (as judged by EGFP fluorescence) were cultured in parallel in the presence and absence of drug and imaged with a Zeiss LSM 510 confocal microscope (Thornwood, NJ). For each compound, we examined ≥3 pairs of ovaries in 2–3 independent experiments.

For live imaging, newborn ovaries were placed in a thin layer of DMEM/10% FBS (Life Technologies) underneath the hydrophilic filter of a Millicell Cell Culture Insert (EMD Millipore) filled with 1.2% agarose/DMEM with 10% FBS (Fig. 1D). The Millicell Cell Culture Insert was then placed in a coverslip-bottomed, 35 mm petri dish with a peripheral ring of 1% agarose (PBS), sealed with parafilm and placed in a 37 °C closed chamber supplied with 5% CO_2_. Ovaries were scanned by confocal microscopy every 10–15 min for up to five days. For each time point, 9–11 stack images were obtained with a Z-voxel equal to 10 µm. Laser power was controlled below 10% and the objective (Plan-Apochromat 20X/0.75) was heated to 37 °C during the experiments. The obtained images were processed by ImageJ [42].

### Electron Microscopy

Normal and *Figla* null newborn ovaries (n = 3) were fixed with 2 % glutaraldehyde in cacodylate buffer pH 7.4, for 2 hr at 4°C. After extensive washing in cacodylate buffer samples were dehydrated through a graded series of ethanol and processed for embedding in LR-White resin. Ultrathin sections were counterstained with uranyl acetate followed by lead citrate and imaged in a Phillips CM120 transmission electron microscope (FEI Company) [18].

### Indirect Immunofluorescence and TUNEL Assay in Ovaries

Histochoice fixed and paraffin embedded sections from newborn mouse ovaries were stained with antibodies to Mvh/DDX4 (1∶100), F4/80 (1∶200) and detected with Dylight coupled goat anti-rabbit (1∶200) and Alexa-466 coupled donkey anti-rat (1∶200) antibodies, respectively. For TUNEL (terminal deoxynucleotidyl transferase dUTP nick end labeling; EMD Millipore) and Mvh double staining, deparaffinized tissue sections were additionally fixed in 1% paraformaldehyde/PBS for 15 min and incubated in 0.1% TritonX-100/10 mM citrate buffer at pH 6.0 for 2 min at 4 °C prior to the TdT reaction. Anti-DIG antibody combined with Alexa466 coupled goat anti-rabbit antibodies were incubated with the sections for 45 min. To quantitate the results, an arbitrary unit was defined as total stained cells from at least 3–6 sections throughout an ovary divided by the total section area (pixels).

### Ovary Histochemistry and Morphometrics

Alternate serial sections (5 µm) of ovaries (2 d/o) were stained with periodic acid-Schiff reagent and hematoxylin. Pyknotic (necrotic or apoptotic) oocytes were scored based on cell size, shape, and condensed chromatin. The total count of such oocytes in ovaries of *Csf1^op/op^* and normal littermates was statistically analyzed by Student's t-test.

## Supporting Information

Figure S1 **EGFP expression in embryonic gonads of** ***Figla-EGFP*** **/** ***Cre*** **transgenic mice.** Female (A–C) or male (D–F) embryonic gonads at E12.5 (A,D), E14.5 (B,E) or E16.5 (C,F) were dissected either from hemizygous *Figla-EGFP*/*Cre* transgenic mice or *Figla-EGFP*/*Cre*; *mTomato*/*mEGFP* mice (insets) and observed by confocal microscopy.
(TIF)

Figure S2 **Macrophage deficiency did not affect oocyte degradation in** ***Figla*** **null ovaries.** (A) A modest reduction of ovarian macrophages was observed in 2 day old *Csf1^op/op^* mice compared to normal littermate control after staining macrophages with F4/80 (green). Ovarian tissue is outlined by a dotted line. (B) P2 ovarian sections from: (1) normal (*Csf1^op/+^*); (2) *Csf1^op/op^*; (3) *Figla* null; and (4) *Csf1^op/op^*; *Figla* null double knockout mice were stained with periodic acid-Schiff and hematoxylin. There was no significant difference in the abundance of oocytes in double knockout and *Figla* null ovaries.
(TIF)

Table S1 **Primer pairs used for mice genotyping.**
(DOCX)

Video S1 **First day culture of normal newborn ovary.** A square indicates somatic cell invasion of oocyte cyst.
(MP4)

Video S2 **Day 2-3.5 culture of normal newborn ovary.** A square indicates somatic cell invasion of oocyte cyst. Arrowheads indicate four growing oocyte, two of which become atretic.
(MP4)

Video S3 **First day culture of Figla null newborn ovary.**
(MP4)

Video S4 **Phagocytes observed in a** ***Figla*** **null ovary.** Arrowheads indicate mobile somatic cells.
(MP4)
