# Correction: *Figla*-*Cre* Transgenic Mice Expressing Myristoylated EGFP in Germ Cells Provide a Model for Investigating Perinatal Oocyte Dynamics

**DOI:** 10.1371/journal.pone.0093347

**Published:** 2014-03-19

**Authors:** The PLOS ONE Staff

Video S1 and Video S3 have been switched in the Supporting Information. Please view the correct videos and their description below.

## Supporting Information

Video S1 **First day culture of normal newborn ovary. A square indicates somatic cell invasion of oocyte cyst.**
(MP4)

Video S3 **First day culture of Figla null newborn ovary.**
(MP4)
