# Supplementary figures and images for: Figla-Cre Transgenic Mice Expressing Myristoylated EGFP in Germ Cells Provide a Model for Investigating Perinatal Oocyte Dynamics
Source: PLoS One. 2014 Jan 6;9(1):e84477. doi: 10.1371/journal.pone.0084477 (PMC3882233; doi:10.1371/journal.pone.0084477)

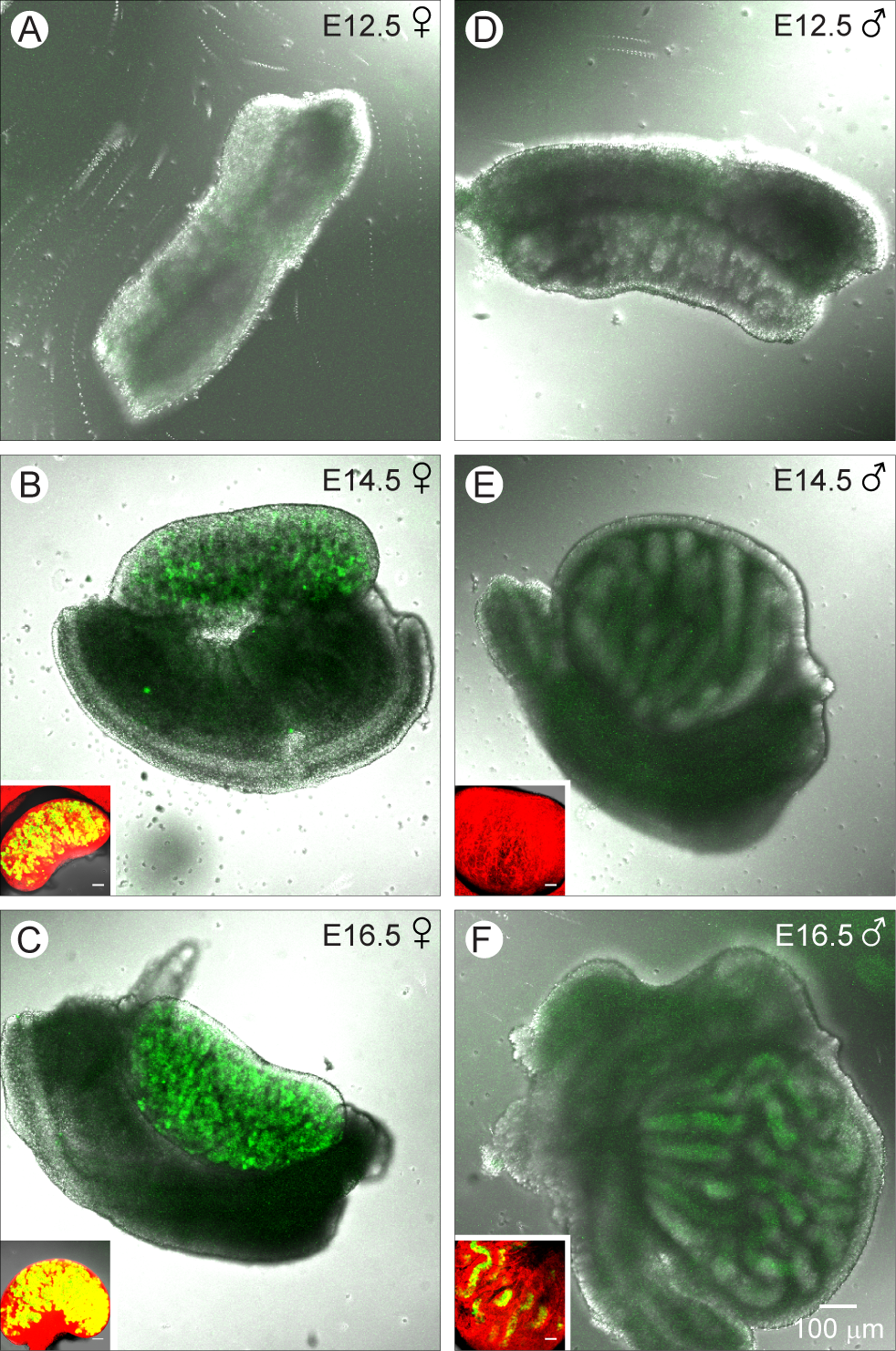

Supplement: Figure S1 — EGFP expression in embryonic gonads of Figla-EGFP / Cre transgenic mice. Female (A–C) or male (D–F) embryonic gonads at E12.5 (A,D), E14.5 (B,E) or E16.5 (C,F) were dissected either from hemizygous Figla-EGFP/Cre transgenic mice or Figla-EGFP/Cre; mTomato/mEGFP mice (insets) and observed by confocal microscopy. (TIF) [file pone.0084477.s001.tif]

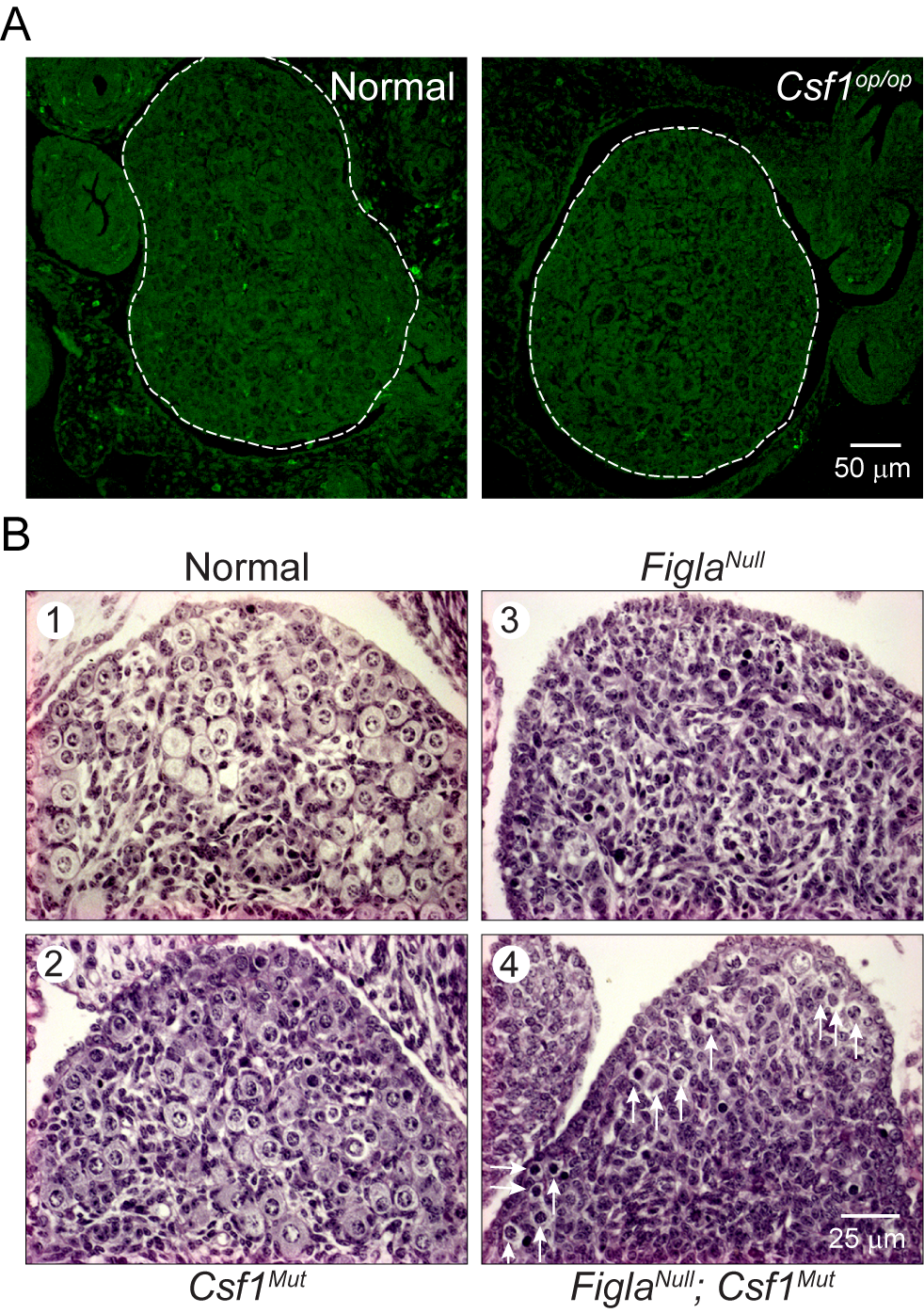

Supplement: Figure S2 — Macrophage deficiency did not affect oocyte degradation in Figla null ovaries. (A) A modest reduction of ovarian macrophages was observed in 2 day old Csf1op/op mice compared to normal littermate control after staining macrophages with F4/80 (green). Ovarian tissue is outlined by a dotted line. (B) P2 ovarian sections from: (1) normal (Csf1op/+); (2) Csf1op/op; (3) Figla null; and (4) Csf1op/op; Figla null double knockout mice were stained with periodic acid-Schiff and hematoxylin. There was no significant difference in the abundance of oocytes in double knockout and Figla null ovaries. (TIF) [file pone.0084477.s002.tif]
