# Supplementary material for: Figla-Cre Transgenic Mice Expressing Myristoylated EGFP in Germ Cells Provide a Model for Investigating Perinatal Oocyte Dynamics
Source: PLoS One. 2014 Jan 6;9(1):e84477. doi: 10.1371/journal.pone.0084477 (PMC3882233; doi:10.1371/journal.pone.0084477)
Supplement: Table S1 — Primer pairs used for mice genotyping. (DOCX) [file pone.0084477.s003.docx]

**Table S1.** Primer pairs used for mice genotyping

| **Mouse line** | **Normal/Mutant** | **Forward primer sequence** | **Backward primer sequence** |
| --- | --- | --- | --- |
| *Figla-EGFP/Cre* | Mutant ^a^ | CACATGAAGCAGCACGACTT | AGTTCACCTTGATGCCGTTC |
| *Figla^-/-^* | Normal | CACCATGGATACAGCACCTG | GGTCATCCGTGGTGGAGTAG |
|  | Mutant ^b^ | CTGGCCCCTTCGTGGTACCACC | CATTTGTCACGTCCTGCACGAC |
| *mTomato/mEGFP* | Normal ^c^ | CTCTGCTGCCTCCTGGCTTCT | CGAGGCGGATCACAAGCAATA |
|  | Mutant ^c^ | CTCTGCTGCCTCCTGGCTTCT | TCAATGGGCGGGGGTCGTT |
| *Csf1^op/op^* | Normal/Mutant ^d^ | TGTGTCCCTTCCTCAGATTACA | GGTCTCATCTATTATGTCTTGTACCAGCCAAAA |

^a^ The amplicon detects EGFP

^b^ [17]

^c^ Genotyping protocol from Jackson Lab

^d^ [41]
